# Facile and scalable production of heterostructured ZnS-ZnO/Graphene nano-photocatalysts for environmental remediation

**DOI:** 10.1038/s41598-018-31539-7

**Published:** 2018-09-07

**Authors:** Sunil P. Lonkar, Vishnu V. Pillai, Saeed M. Alhassan

**Affiliations:** Department of Chemical Engineering, Khalifa University of Science and Technology, P.O box 127788, Abu Dhabi, UAE

## Abstract

A facile and eco-friendly strategy is described for the synthesis of ZnS-ZnO/graphene heterostructured nano-photocatalysts for the first time. This solvent-free and technologically scalable method involves solid-state mixing of graphite oxide (GO), Zn salt and surfeit yet non-toxic elemental sulfur using ball-milling followed by thermal annealing. The as-formed hybrids are composed of uniformly distributed *in-situ* formed ZnS-ZnO nanoparticles simultaneously within the thermally reduced GO (graphene) matrix. A series of hybrid compositions with varying content of ZnS/ZnO and graphene were prepared and thoroughly characterized. Further, the effect of heterostructure composition on the photocatalytic properties was investigated under visible-light illumination. The synergistic ZnS-ZnO/graphene hybridization promoted the band-gap narrowing compared to the pristine ZnS nanoparticles. The ZnS:ZnO composition was controlled by graphite oxide under thermal treatment and observed to be a crucial factor in enhancement of photocatalytic activity. As a proof of concept, the phase optimized and surface enhanced ZnS-ZnO/graphene nano-photocatalysts was tested towards visible light driven photocatalytic degradation of environmentally harmful organic dyes and toxic phenol molecules from aqueous media. The presented cost-effective strategy provides high potential in large-scale production of heterostructured nano-photocatalysts for environmental remediation and photocatalytic greener production of hydrogen.

## Introduction

Due to the ever-growing environmental concerns, the quest for highly active and low cost yet visible light responsive photocatalyst for effective environmental remediation is still ongoing and emerged as an important thrust area of research^1–3^. So far, semiconductor based nanostructured materials have attracted great deal of interests as a potential photocatalyst^4–6^. However, due to poor absorption of light and fast recombination of photogenerated electrons and holes, these individual semiconductors further needs fine-tuning in order achieve unique band gaps that can effectively utilize light energy for oxidative degradation of toxic organic compounds and pollutants. Hence, nanoscale hybridization of individual photocatalysts with hierarchical structure and dimensions offers innovative and efficient strategy to fabricate photocatalyst with enhanced photo-response and deprived electron-hole recombination^5,7,8^. Therefore, wide arrays of coupled semiconductor based heterostructured nanohybrids were synthesized and used for various photocatalytic transformations^9–11^. However, most of them are fancy and complex ternary chalcogenide compounds based photocatalysts which display a strong visible-light absorption and narrow band gaps, but their production complexity and high-cost is still a major roadblock from an industrial application perspectives^12^. Hence, in this context, the nanostructured hybrids composed of conventional photocatalysts such as ZnS and ZnO with wide range band gap (Eg = 3.54 and 3.37 eV at 300 K, respectively) are still considered as the most attractive photocatalyst option mainly because of its exceptional optical properties, environmental sustainability and cost-effectiveness^13–15^. It has been demonstrated that the heterostructure combination of both ZnO and ZnS are better in realizing the photocatalytic decomposition of the organic pollutants over the individual components^16^. Also, the band gap of these nanoparticles can be narrowed to visible region by creating ZnS-ZnO heterostructures^17^. The typical synthesis methods that are commonly used in the preparation of such hybrid photocatalysts comprise wet-chemical synthesis^18,19^, co-precipitation method^20^, thermal and hydrothermal methods^21–23^, also sulfurization of ZnO with noxious H_2_S gas at high temperatures was also practiced^24,25^. Furthermore, most of these processes are highly intricate and uses toxic agents, which can restrict their large-scale productions. Hence, the development of inexpensive, scalable and environment friendly method to prepare such heterostructured nanohybrids is the key to the commercialization of photocatalysts for waste water treatment and other environmental remediation applications.

Furthermore, surface immobilized photocatalysts are often exhibit significantly higher remediation due to internal efficiency related to separation, and establish affinity towards external contaminant molecules etc^26,27^. Recently, Ma *et al*.^20^ has successfully demonstrated the formation of ZnO-ZnS/carbon hybrids as an efficient photocatalyst in dye removal. In this context, in addition to its size controlling effect and surface area enhancing activity, if support material can also contribute to the photocatalytic activities of such heterosructures then that could be an interesting photocatalyst composition. For example, the hybridization of individual ZnS and ZnO nanoparticles with graphene is known to prevent the nanoparticles from aggregating due to its layered structures and high surface area and uniform nanoparticle distribution can be achieved^28,29^. These nanohybrids showed improved photocatalytic characteristics due to the electron detention and its transferability within graphene that effectively prevent the recombination the photo-excited charge carriers i.e electrons and holes^16^. Also, graphene demonstrated as macromolecular sensitizer that can transform ZnS from wide band gap semiconductor to the visible light photocatalyst^30^. Thus, the incorporation of graphene together with semiconductor nanocomposites can significantly enhance the photocatalytic performance in degradation of dyes and other organic pollutants^31^. Hence, we believe that hybridizing ZnS-ZnO heterostructures with graphene could further enhance the photoactivity of the resulting photocatalyst under visible light. The addition of GO provides dual role, first it provides valuable support to nucleate nanoparticle precursors and later during thermal annealing process, the liberated oxygenated species can effectively oxidize some of the ZnS into ZnO.

In the present research work, we report a simple, scalable and environment friendly approach for the first time to fabricate nano-photocatalyst hybrids composed of ZnS-ZnO and graphene. The formation mechanism of ZnS-ZnO and simultaneous reduction of GO was explained according to a series of experiments and structural characterizations. The photocatalytic efficiency, adsorption performance and recyclability of the nanohybrids is estimated by monitoring the most prominent industrial water contaminants i.e organic dyes such as cationic (methylene blue, MB), anionic (methyl orange, MO) and toxic nitro-phenols (2-NP and 4-NP) as model pollutants. It is demonstrated that the hybridization of ZnS-ZnO heterostructure and graphene can potentially lower the rate recombination of photo-generated charge carriers and exhibit a significant photocatalytic activity and efficiency.

## Result and Discussion

**Figure 1:**
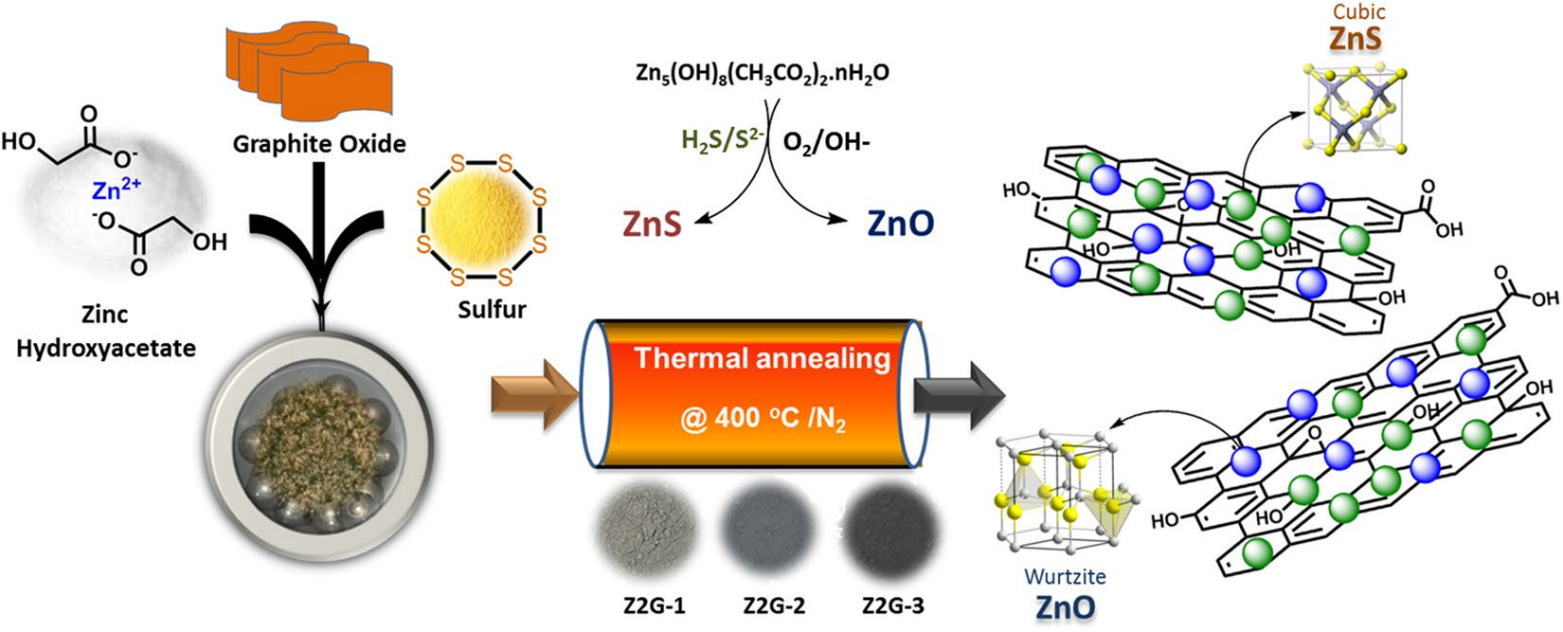
Schematic illustrations for the preparation of the ZnS-ZnO/graphene nanohybrids using solid-state thermal synthesis method.

The formation mechanism of the as-prepared ZnS-ZnO/graphene composites prepared via solid-state synthesis is shown in Fig. 1. In this study, zinc hydroxyacetate was used as the source of zinc, surfeit and non-toxic elemental sulfur as a sulfur source and graphite oxide as graphene precursor. During the ball milling process, the zinc hydroxyacetate and sulfur was homogenized through intercalation into layered GO. Then, under thermal treatment Zn^2+^ simultaneously reacts with *in-situ* formed reactive sulfur and oxygenated species liberated through thermal decomposition of both Zinc salt and GO to form spherical ZnS and ZnO nanocrystals under different stoichiometric conditions. The surface of the thermally reduced GO (graphene) sheets was expected provide support for ZnS/ZnO nucleation sites, resulting in a uniform dispersion of nanosized heterostructured particles onto graphene. Evidently, the color change was observed from brownish to grey and become darker with increasing the graphene content.

**Figure 2:**
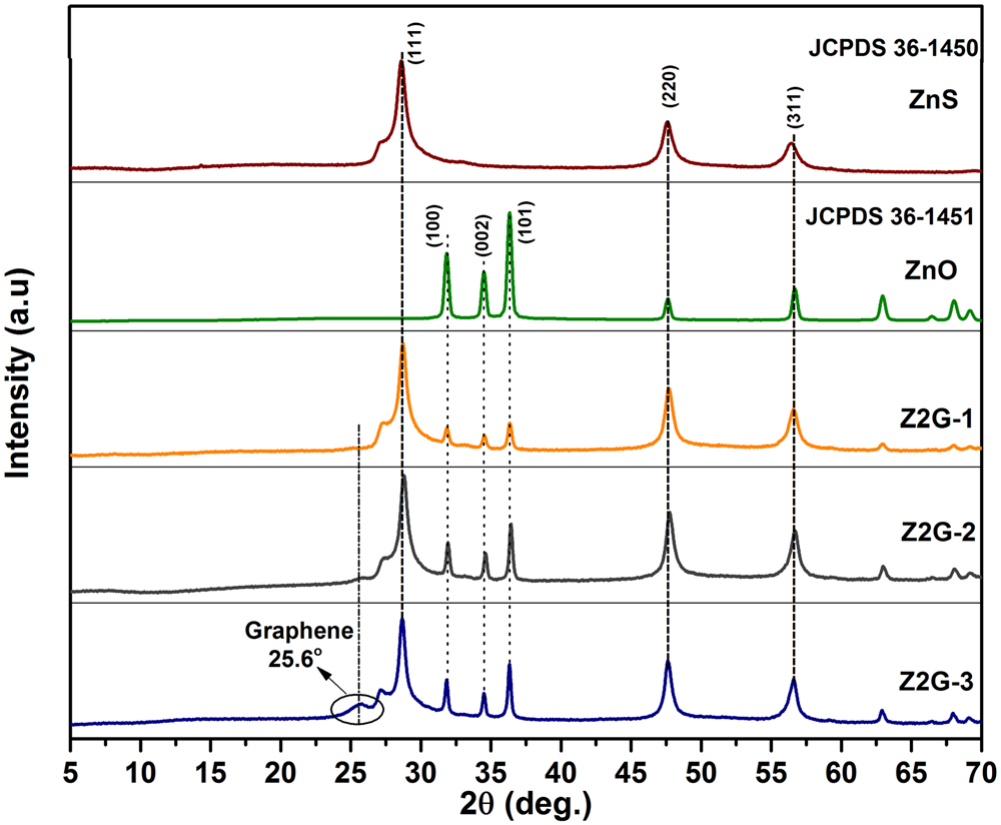
XRD diffraction patterns of ZnS (**a**), ZnO (**b**), Z2G-1 (**c**), Z2G-2 (**d**) and Z2G-3 (**e**).

The crystalline structures and phase orientations of ZnS-ZnO/graphene nanostructured hybrids are examined by PXRD analysis (Fig. 2). For ZnS, the peaks at 28.59°, 47.62°, and 56.47° were recorded which can be indexed to the (111), (220), and (311) planes of the cubic ZnS (JCPDS 36-1450). Similarly for ZnO, the sharp peaks at 31.83°, 34.48°, 36.32°, 47.62°, 56.76°, and 62.93° indicated the existence of a wurtzite phase of as formed ZnO structure (JCPDS No. 36-1451). After thermal treatment of ball milled homogeneous mixture of zinc salt, sulfur and GO, the characteristic peaks similar to ZnS and ZnO nanoparticles are found in the XRD patterns of resulting nanostructured hybrids (Fig. 2c–e). In addition, an appearance of a new broad peak around 25.57° was solely indicated the characteristic (002) planes of the graphene (Fig. S1), which gradually intensify with graphene loading. The disappearance of the characteristic peaks assigned to graphite oxide around 2θ = 9.7° in Z2G composites indicated the successful thermal reduction of the layered GO into graphene. It has been also observed that the loading of GO played significant role in the formation of both ZnS an ZnO phases in the resulting Z2G hybrid. For Z2G-3 hybrids, intensity of the peak corresponding to the ZnO phase was gradually increases in compared to Z2G-1, which is attributed to the increasing amount of oxidative GO in the starting composition. These results confirm that the GO offers more oxygenated species in order to oxidize the ZnS in to ZnO and eventually control the ZnS:ZnO ratio in the final nanohybrid composition which is proportional with GO content (Fig. S2). Further, the absence of any other dominant peaks in resulting XRD pattern confirms that the Z2G nanohybrids were exclusively made up of ZnS, ZnO and graphene phases. Furthermore, the average crystallite size of ZnS and ZnO in resulting Z2G nanohybrids was estimated using Debye-Scherrer’s equation (1)^32^:

1 $${\rm{D}}=\frac{{\rm{K}}{\rm{\lambda }}}{{\rm{\beta }}\,\cos \,{\rm{\theta }}}$$

where D is average crystallite size (nm); K dimensionless shape factor, with value of 0.89; λ represent X-ray wavelength; β stands for the line broadening at half the maximum intensity and θ is the Bragg angle. The calculated crystallite size is presented in Table 1.

**Table 1:** Crystallite size of ZnS and ZnO nanoparticles in resulting Z2G nanohybrids.

Sample	ZnS Average Dp (nm)	ZnO Average Dp (nm)
ZnS	13.89	—
ZnO	—	27.67
Z2G-1	7.89	22.61
Z2G-2	6.87	20.24
Z2G-3	6.38	17.46

It can be seen that the particle size of ZnS is significantly decreased with increasing the content of graphene and similar trend was observed for ZnO nanoparticles. These results underline that role of graphene nanosheets which can act as an excellent support in controlling the size of these *in-situ* formed ZnS-ZnO heterostructured nanohybrid particles.

**Figure 3:**
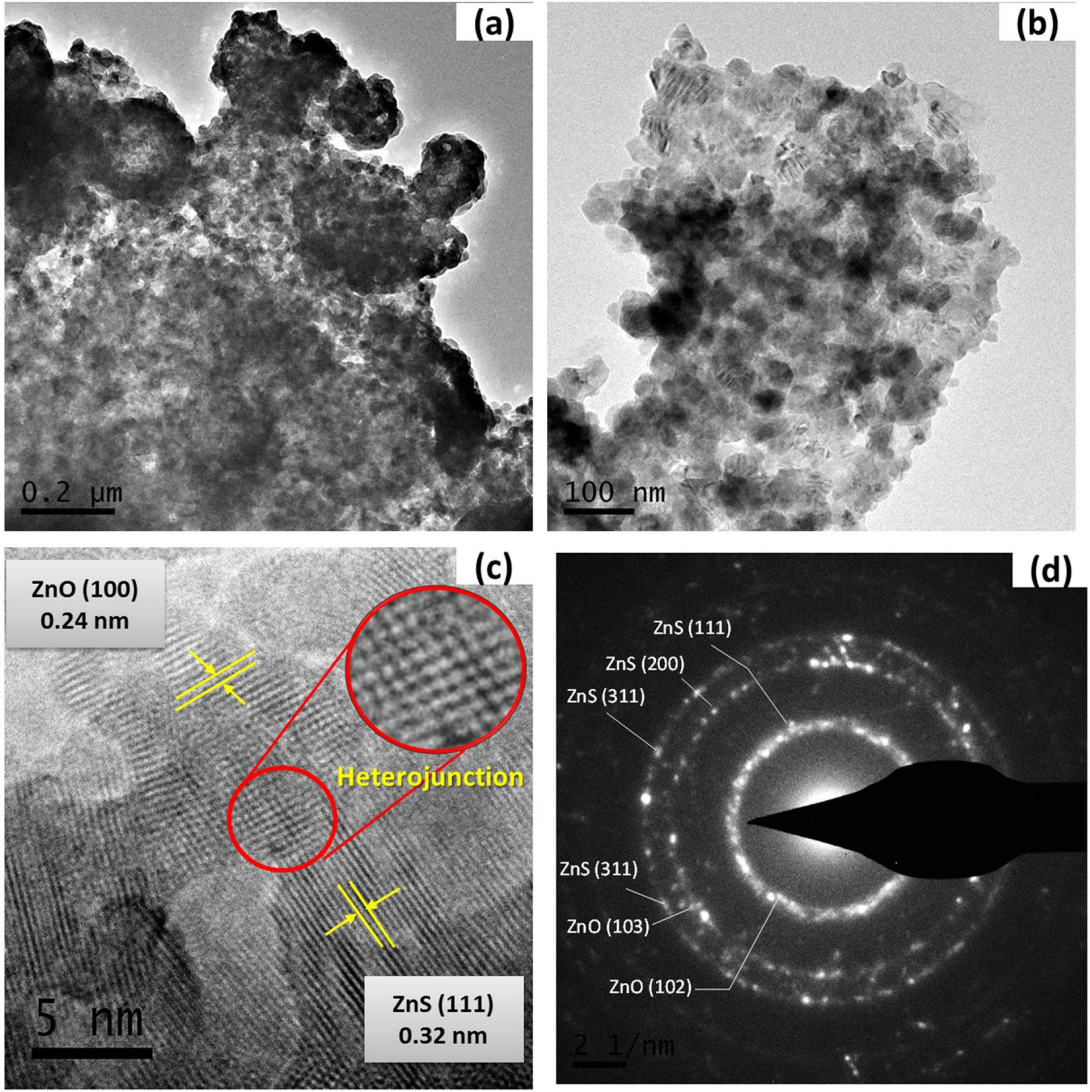
Low-magnification TEM image (**a**,**b**), high-resolution TEM images (**c**), and SAED pattern (**d**) of Z2G-1 nanohybrid.

The morphological and structural evolutions during the *in-situ* synthesis of these as-prepared nanohybrids were investigated by TEM and SEM measurements. Figures 3a,b and S3 represents the typical TEM image of a Z2G-1 hybrid, which indicates the *in-situ* formed ZnS-ZnO nanoparticles (ca. 5–20 nm) are uniformly distributed and tightly aggregated on the graphene support was observed. The HRTEM micrograph (Fig. 3c) indicated the interface region of a characteristic heterojunction, and the typical lattice fringes of the nanoparticles. The characteristic lattice fringes of the ZnS nanoparticles having lattice spacing of 0.32 nm for (111) planes are well intermixed with lattice fringes of the ZnO having the lattice spacing of 0.24 nm for (101) planes were observed. This observation clearly reveals formation of heterostructured nanohybrids composed of cubic blende ZnS intermixed wurtzite ZnO and graphene^33^. Besides, the SAED pattern (Fig. 3d) displayed a set of well-defined diffraction rings which indicates the existence of highly crystallized ZnS-ZnO nanoparticles in the resulting nanohybrids, which is in agreement with the XRD result shown in Fig. 1.

**Figure 4:**
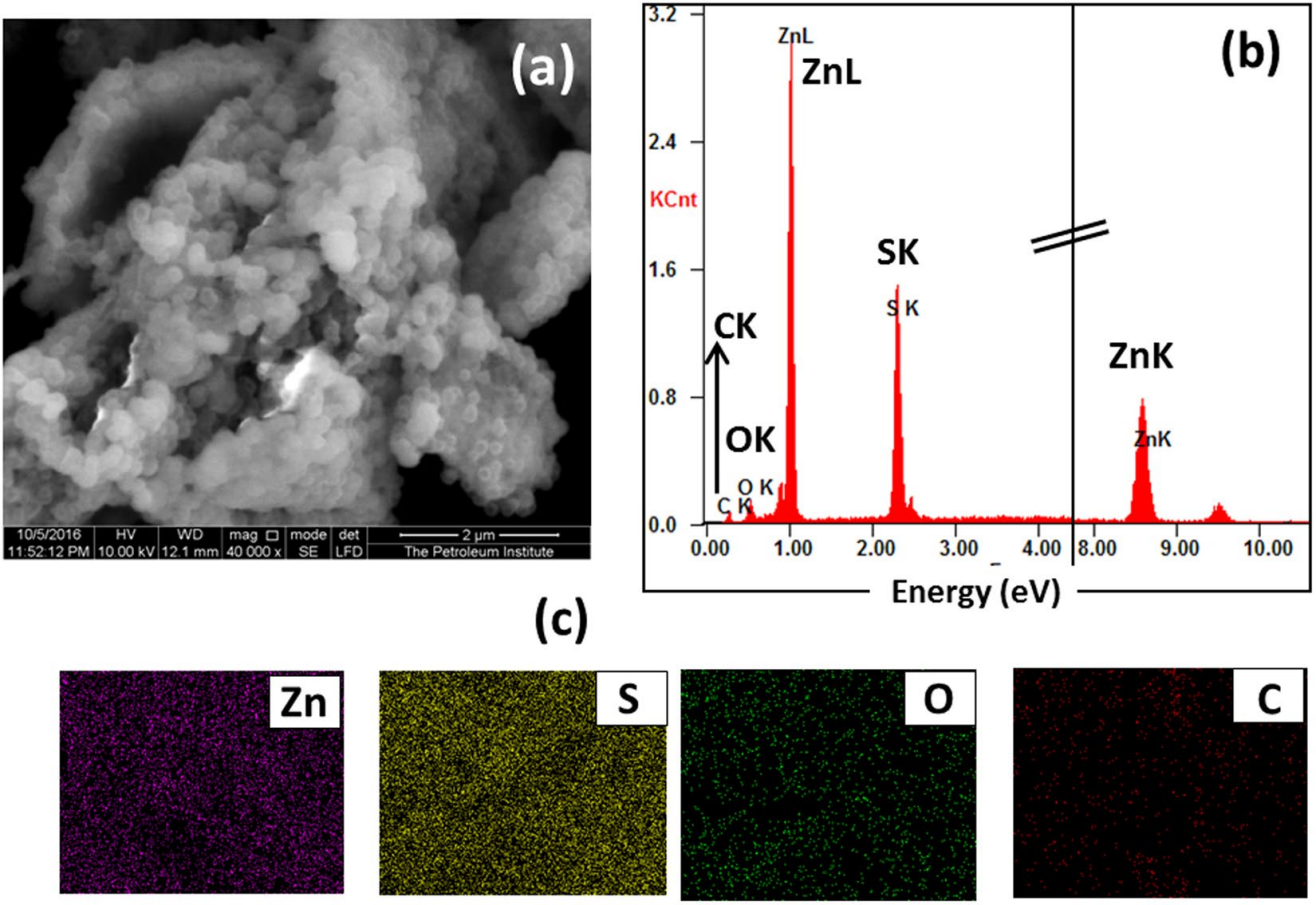
SEM image of the as-prepared Z2G-1 nanohybrid (**a**), EDS spectrum (**b**) and corresponding elemental mapping images for Zinc, Sulfur, Oxygen and Carbon (**c**), respectively.

The SEM in conjunction with energy dispersive spectroscopy (EDS) gives more insight on the morphological features and the elemental composition of the as prepared Z2G hybrids and same were showed in Fig. 4 for Z2G-1 sample. It can be clearly seen that the ZnS-ZnO heterostructure with spherical assemblies were uniformly spread on the surface of the wrinkled micro sheets 2D graphene (Fig. 4a). The EDS spectrum of Z2G-1 hybrid (Fig. 4b) shows the corresponding elemental composition indicating Zn, S, O and C elements which further confirmed heterostructured hybrid formation, composed of ZnS-ZnO and graphene. Further, the elemental mappings showed in Fig. 4c confirm uniform distribution of the ZnS-ZnO species supported by graphene. Similarly, the other hybrids (Z2G-2 and 3) also showed similar morphological features but the increase in graphene content cause more crumpling and agglomerated sheet like morphology in compared to pristine ZnS nanoparticles spherical morphology (Fig. S4).

The decreased S/O ration with increase in GO content for Z2G-2 and 3 further confirmed the higher conversion of ZnS into ZnO under oxidative thermal treatment. In summary, ZnS-ZnO heterostructures have been *in-situ* formed and deposited on the graphene nanolayers which significantly changed the morphology and the ratio of ZnS-ZnO heterostructure in the final nanocomposites. Overall, both TEM and SEM investigations confirms the formation of good interfacial contact between ZnS-ZnO nanostructures and graphene sheet due to synergetic effect between individual components. Such interfacial interactions are critical from photocatalysis perspectives considering the improved charge transfer process between graphene and semiconductor nanoparticles^34^.

**Figure 5:**
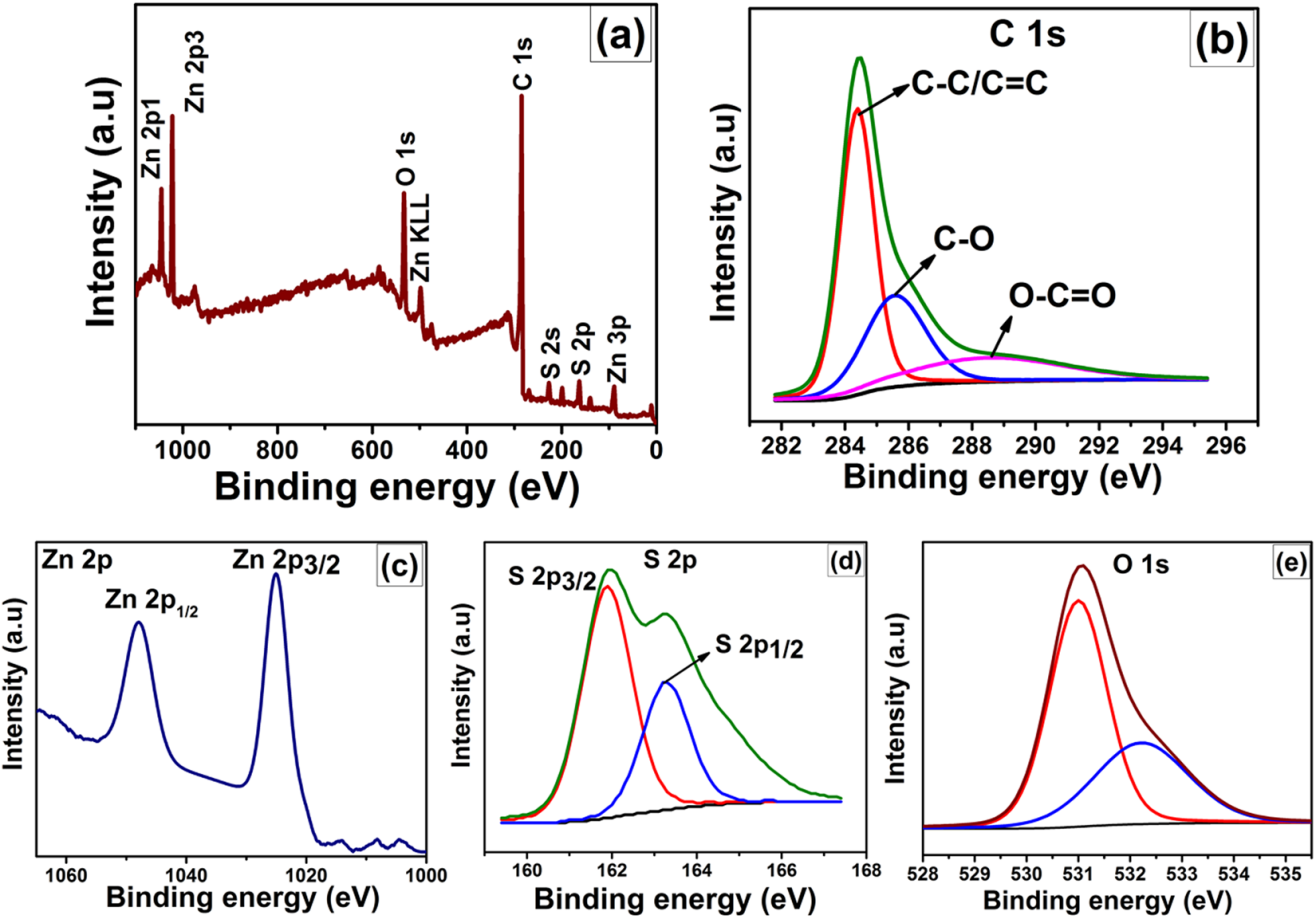
XPS spectroscopy of Z2G-1 nanohybrid (**a**) survey spectrum; high resolution scan of (**b**) carbon 1 s, zinc 2p (**c**) and sulfur 2p (**d**).

The chemical composition and the binding energy state of the resulting Z2G heterostructured nanohybrids were determined by XPS measurements. As showed in Fig. 5a, the XPS survey spectra of the Z2G-2 sample clearly indicates the characteristic peaks of Zn2p, S2p, C1s, and O1s and absence of any other element peak confirms pure state of the as formed ZnS-ZnO nano-assemblies within graphene layered network. The high resolution C1s spectrum (Fig. 5b) shows three characteristic peaks with binding energies of 284.4, 285.5, and 289.9 eV that are attributed to the carbon atoms such as delocalized sp2 -hybridized C–C/C=C, C–O, and O–C=O groups, respectively^35^. The decrease in these peak intensities compared to GO (Fig. S5) further indicates that the thermal reduction of the GO during the formation of Z2G nanohybrids under thermo-annealing process. The reduction of GO to graphene is known to develop the high electron mobility, necessary for the enhancement of photocatalytic process^36^. The Zn 2p spectrum displays characteristic peak at 1022 eV, attributed to the Zn 2p_3/2_ spin orbit peaks of the ZnS and ZnO phase^28,37^. The presence of ZnS can be further confirmed by the S2p spectrum (Fig. 5d) with the characteristic peaks at 161.8 and 163.1 eV, which indicate the S2p_3/2_ and S2p_1/2_ peaks in the ZnS phase^37^. Similarly, Fig. 5e showed the high resolution O1s peak for Z2G-1, which can be fitted into two peaks. The lower energy peak located at 531.0 eV corresponded to the oxygen atoms coordinated with Zn atoms in ZnO lattice. On the other hand, the higher binding energies located at 532.5 eV in Z2G nanohybrid could be due to the presence of hydroxyl groups (O hydroxyl) and other oxygen functional groups coming from graphene, respectively^28^. Therefore, the XPS results indicated the ZnS particles formed on the graphene surface with some oxygen-containing groups corresponds to the existence of ZnO particles.

**Figure 6:**
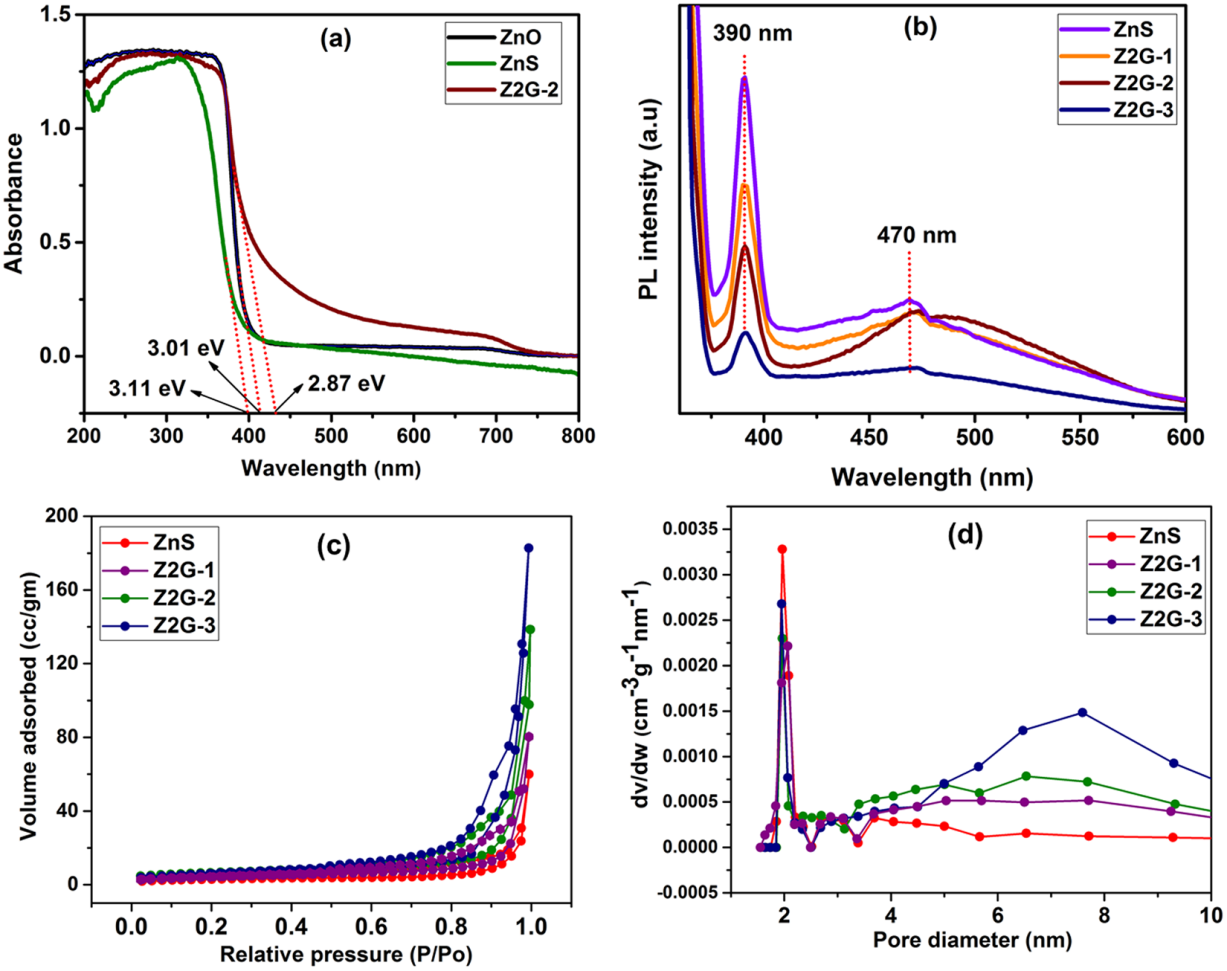
UV-Vis absorbance spectra (**a**) and photoluminescence spectra (**b**) of Z2G nanohybrids and ZnS nanoparticles. Nitrogen adsorption–desorption isotherms (**c**), and BJH pore diameter plots (**d**) for Z2G nanohybrids.

The UV-visible diffuse reflectance converted absorbance spectrum was recorded to evaluate the optical properties of the resultant Z2G nanohybrids and pristine ZnS/ZnO nanoparticles as shown in Fig. 6a. It was noted that the addition of graphene extends nanohybrid absorbance into the visible region, which was further supported by color change of the sample (in-set Fig. 1). The band gap energy measured by employing Kubelka-Munk function showed band gap around 3.11 eV and 3.01 eV for ZnS and ZnO nanoparticles, which is narrower than their bulk counterparts^38^. In the graphene containing samples, Z2G-1 and Z2G-2 shows band gap of 2.96 eV and 2.87 eV, respectively. Due to high background absorption we could not able to measure the band gap for Z2G-3 sample. These findings indicated that the band gap energy of the resulting ZnS-ZnO heterostructure was lowered when hybridized with 2D graphene at nanoscale which in agreement with previous reports for graphene nanohybrids such as ZnS-graphene^30^ and TiO_2_-graphene^39,40^. Overall, the improved optical properties for the resulting ZnS-ZnO/graphene heterostructured hybrids would be beneficial for visible-light harvesting and photocatalysis applications.

The prospects of the photo-induced electron–hole pair recombination was further investigated using photoluminescence (PL) spectra at an excitation wavelength of 350 nm (Fig. 6b). The PL spectra of ZnS nanoparticles and Z2G hybrids show a sharp blue emission peak around 390 nm. The intensity of UV emission at 390 nm is related to the electron and hole pair recombination in the conduction and valence band, respectively^41^. In addition, the blue-green emissions in the range between 450 to 550 nm (visible region) were observed, which can be attributed to the sulfur vacancies and interstitial lattice defects. Further, the green emission is owing to the self-activated defect centers related to Zn-vacancies present in ZnS nanoparticles^42^. It was observed that the PL peak intensities for Z2G hybrids are significantly lowered with increase in graphene content which also implies the subsequent increment in the ZnO content. Such quenching implies that an active interfacial charge-transfer process occurred between the photo-excited ZnS-ZnO nano-assemblies and the graphene sheets. This also paves an additional pathway for disappearance of the charge carriers during the photo-excitation process. Further, the high electron affinity of graphene can attract more photo-excited electrons from the ZnS-ZnO heterostructure; such electron acceptor-donor combination could lead to an energetic electron transfer from ZnS to Z2G-3 and can reduce possible electron–hole pair recombination effectively.

Figure 6c shows the nitrogen gas adsorption-desorption isotherms of the resulting ZnS and ZnS-ZnO/graphene nanohybrid samples. According to the IUPAC classification, all of these isotherm patterns can be classified as near type IV isotherm which is characteristic of the inhomogeneous mesoporous materials having H1 hysteresis loop. The BJH pore size distribution curves of the nanohybrids are shown in Fig. 6d. The measured BET surface areas for ZnS and ZnS-ZnO/graphene nanohybrids are presented in Table 2. After hybridization, the surface areas tend to increase with increase in graphene content, this further evidence the *in-situ* formation of nanostructured hybrids. Mesoporosity in the resulting nanohybrids were further confirmed from the pore size distribution peaks that are centered around a 2 nm and a broad peak around 7.6 nm^43^. The total volume of pores for nanohybrids was between 0.094 to 0.254 cm^3^g^−1^, respectively. Both the BET surface area and the average pore size of the ZnS-ZnO/graphene hybrids are larger than those of pure ZnS, which indicates that the resulting nanohybrids can provide a larger interface to enhance the contact between the dye and the active catalyst and facilitate photocatalytic reaction.

**Table 2:** The BET surface area, BJH pore diameter and pore volume properties of the pure ZnS and Z2G nanohybrids.

Sample	BET surface area (m^2^/g)	Pore diameter (nm)	Pore volume (cm^3^/g)
ZnS	10.69	1.86	0.094
Z2G-1	25.29	2.06	0.130
Z2G-2	64.4	1.9	0.152
Z2G-3	85.3	1.89	0.254

The loading of ZnS and ZnO in the final ZnS-ZnO/graphene heterostructured nanohybrids under different graphite oxide concentrations were summarized in Table 3. As the concentrations of stoichiometric amount of GO used to obtain graphene was increased from 11.1 to 66.9 wt%, the weight percent of ZnS decreased from 90.88 to 56.17 wt-%. Similarly, relative to ZnS, the ZnO concentration in hybrid was increased from 4.11 to 15.54 wt-%. These results indicated that the higher GO amount used as graphene precursor is oxidizing the ZnS in to ZnO during *in-situ* process to obtain ZnS-ZnO heterostructure and thermal reduction of GO simultaneously.

**Table 3:** The elemental composition of ZnS and ZnO in the resulting Z2G nanohybrids.

Samples	GO:TRG (wt%)	^#^S (wt%)	Zn (wt%)*	ZnS (wt%)	ZnO% relative to ZnS	Graphene
ZnS	0	32.8	67.23	100	0	0
Z2G-1	11.1:5	29.59	65.25	90.88	4.5	5
Z2G-2	22.2:10	24.5	63.79	74.46	20.87	10
Z2G-3	66.9:30	18.48	49.71	56.17	24.62	30

^#^S concentration measured by CHNS elemental analysis and ^*^Zn concentration is measured by XPS measurements.

Raman spectroscopy was also employed to investigate the crystal structure of ZnS-ZnO-graphene nanohybrids (Fig. S6). For Z2G-1, Raman peaks appear at 212 cm^−1^ and 329 cm^−1^ corresponding to the characteristic E_1_ (TO) and A_1_ (LO) modes of the ZnS^44^. A blue shift in both E_1_ and A_1_ energy modes for Z2G-1 in compared to the bulk ZnS (E_1_: 274 cm^−1^ and A_1_: 352 cm^−1^) was observed which can be attributed to the confinement effect of optical phonons in the ZnS nanoparticles^45^. Similarly, Raman bands at about at 425 cm^−1^ and 560 cm^−1^ assigned to A_1_ (TO) and E_2_ (high) vibration modes of wurtzite ZnO were observed^46^. The increase in the intensity of ZnO peaks in Z3G hybrids can be ascribed due to the oxidation ZnS under high power laser. Additionally, graphene exhibit two main peaks attributed to the D-band at 1354 cm^−1^ evolved from distortion of sp^2^ structure by creation of sp^3^ carbon centers, whereas the G-band occurred at about 1589 cm^−1^, is due to E_2g_ mode and arise from the stretching of the C-C bond in the graphitic materials.

**Figure 7:**
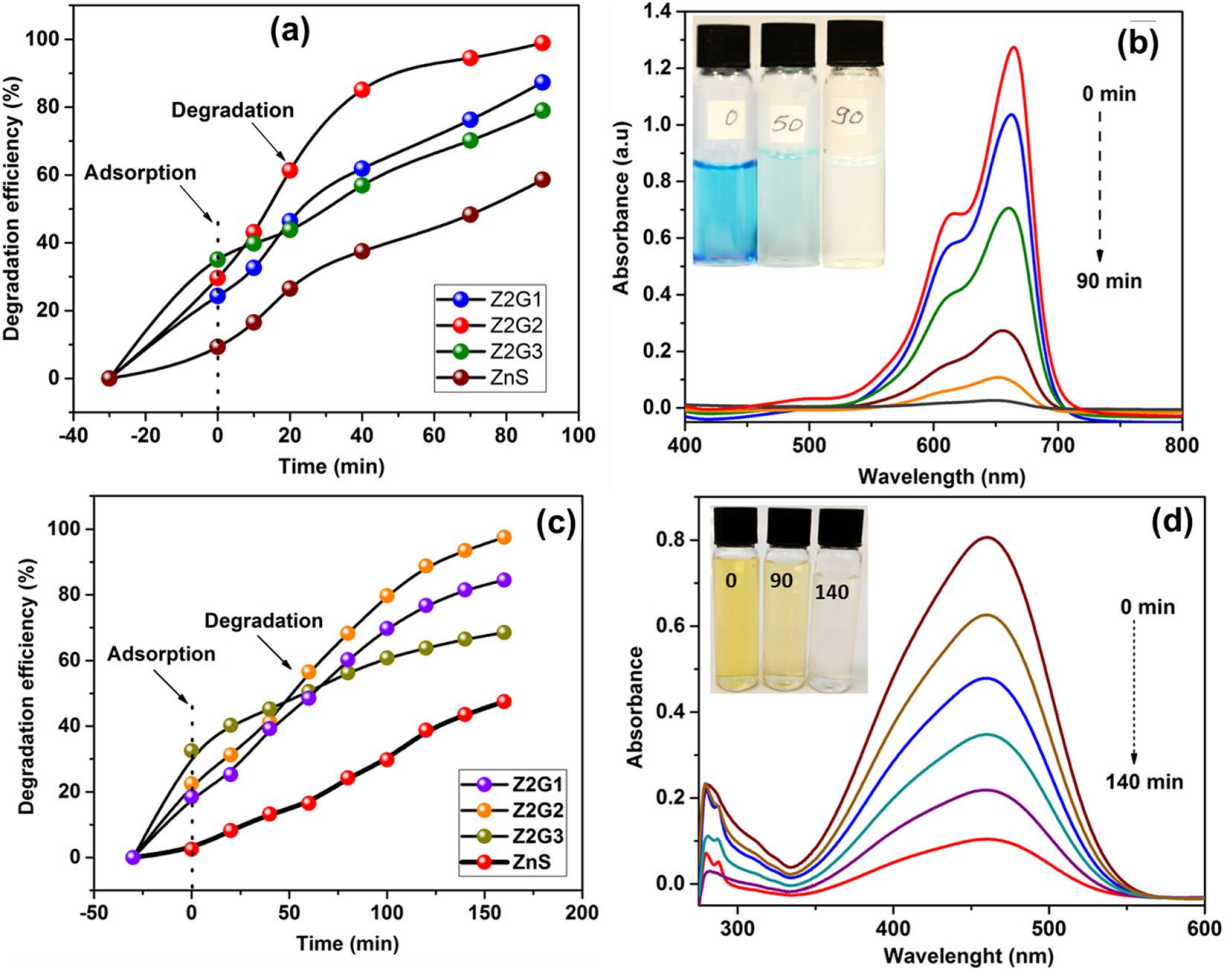
Photocatalytic degradation efficiency of Z2G nanohybrids towards MB (**a**), MO (**c**) dyes. UV-Vis absorption spectrum of Z2G-2 nanohybrid at different time intervals (**b**) MB, (**d**) MO dyes (in-set discoloration of dyes).

Owing to its tunable band structure, the resulting Z2G nanohybrids are expected to display enhanced photo-catalytic properties. Hence, the visible-light driven degradation of harmful dyes such as methylene blue (MB) and methyl orange (MO) and toxic nitro-phenols (2-NP and 4-NP) were carried out as performance assessment test for synthesized nano-photocatalysts and showed in Fig. 7. First, the MB and MO dye adsorption and photocatalytic performance of Z2G nanohybrids and ZnS nanoparticles was tested (Fig. 7a–d). Since the Z2G nanohybrids are highly porous and having more surface area over pristine ZnS nanoparticles, it was expected that hybrids can exhibit characteristic dye adsorption phenomenon. Hence, to monitor the adsorption characteristic of the resulting nanohybrids, the adsorption measurements the dark were also performed. It was observed that the Z2G hybrids shows higher equilibrium adsorption capacity compared to ZnS which exhibit smallest capacity. Amongst Z2G hybrids, the adsorption capacity for both the dyes increases with increasing graphene content and which is due to increased micropore volume and the higher surface area (Table 2). Further, the visible light driven photocatalytic degradation of dye was studied at different time intervals. The MB dye degradation efficiency of the photocatalysts was measured to be around 99%, 87%, 79% and 59% using the Z2G-2, Z2G-1, Z2G-3 and ZnS photocatalysts, respectively under 90 minutes of irradiation (Fig. 7a). Similarly, for MO the dye degradation efficiency of the photocatalysts was measured to be 97.5%, 84.5%, 68.5% and 47% for Z2G-2, Z2G-1, Z2G-3 and ZnS photocatalysts, respectively for 160 minutes of light irradiation (Fig. 7c). The superior photocatalytic activity of the Z2G-2 nanohybrids in both the cases was ascribed due to the several comprehensive factors. First, the synergy between ZnS-ZnO heterostructure and smaller crystallite size of both ZnS and ZnO due to introduction of graphene which implies that Z2G possesses resilient oxidation-reduction capacity owing to the quantum-size effect^47^. Second, the improvised surface properties of the nanohybrid could offer abundant active sites required for efficient light harvesting, resulting into improved photocatalytic activity^36^. Third is the integration of the graphene that could lead to the significant reduction of electron–hole pair recombination through photosensitization. Furthermore, among the Z2G nanohybrids, a higher photocatalytic performance was noted for Z2G-2 sample which could be attributed to the optimized composition of photocatalytically active ZnS-ZnO heterostructure and graphene in Z2G sample. Also, a relatively higher surface area, which was expected to offer more sites for the photocatalytic activity. The lower photocatalytic activity was noted for Z2G-3 because the potential catalytically active sites can be clogged due to the higher graphene content, thereby shielding of the incident light that can reduces the light absorption by ZnS-ZnO photocatalyst^36^. Also, using Z2G-2 photocatalyst the spectral peak intensity of MB dye at 664 nm (Fig. 7b) and MO dye at 460 nm (Fig. 7d) was gradually gone down with increasing irradiation time indicating the photodegradation of the dyes. Further, the inset images indicated that upon given visible light irradiation time, a complete dye discoloration was also achieved.

The photocatalytic activities of the nanohybrids were further analyzed by studying kinetics of photocatalytic degradation using Langmuir–Hinshelwood model^48^:

2 $$r=\frac{{\rm{d}}C}{{\rm{d}}t}=\frac{{k}_{1}{k}_{2}C}{1+{k}_{2}C}$$

where r is the degradation rate of the reactant (mg L^−1^ min^−1^), C is the concentration of the reactant (mg L^−1^), k_1_ is the reaction rate constant (mg L^−1^ min^−1^), k_2_ is the adsorption constant of the reactant (L mg^−1^). When C is very small, the equation can be simplified to:

3 $$\mathrm{ln}\,\frac{{C}_{0}}{C}={k}_{1}{k}_{2}t=Kt$$

where C_0_ is the initial concentration of the reactant and K_t_ is the first-order rate constant obtained from the slope of the graph of ln(C_0_/C) vs. time (t). Figures S7 and S8 shows the plots of ln (C_0_/C) vs. time for Z2G nanohybrids and ZnS NPs, which resulted into straight lines confirming the degradation of both MB and MO, follows the first-order kinetic reaction behavior. The MB dye photodegradation rate constants (K) for the Z2G-1, Z2G-2, Z2–3 and ZnS NPs samples were measured to be 0.012, 0.024, 0.009 and 0.005 min^−1^, respectively. The Z2G-2 nanohybrid showed about 5 times highest K value compared to the ZnS NPs and 1.5 times of Z2G-3 hybrids, respectively. Hence, the higher K value indicates a higher photocatalytic dye degradation rate for Z2G-2 nanohybrid. For MO, the estimated K values are 0.011 (Z2G-1), 0.019 (Z2G-2), 0.009 (Z2G-3) and 0.006 (ZnS) min^−1^, further signifies the higher photocatalytic activity for Z2G-2 nanohybrids. As discussed earlier, the higher K values can be attributed to the optimized composition of *in-situ* formed and graphene wrapped nanosized ZnS-ZnO assemblies, reduced recombination of charge carriers, and band gap narrowing. Similar to the enhanced rate of photodegradation and superior kinetics, the recyclability of a given photocatalyst in the course of photocatalytic reaction is equally important and considered as a critical factor from large scale practical application perspectives. The recyclability tests were therefore performed by repetitive photocatalytic reaction using Z2G-2 photocatalyst under visible light irradiation. It has been observed that after 5 cycles of repeated use, the decrease in photocatalytic efficiency was merely about 6.3% for MB and 8.5% for MO was observed (Fig. S9). These findings revealed the significant stability of the resulting heterostructured Z2G nanohybrids photocatalyst.

**Figure 8:**
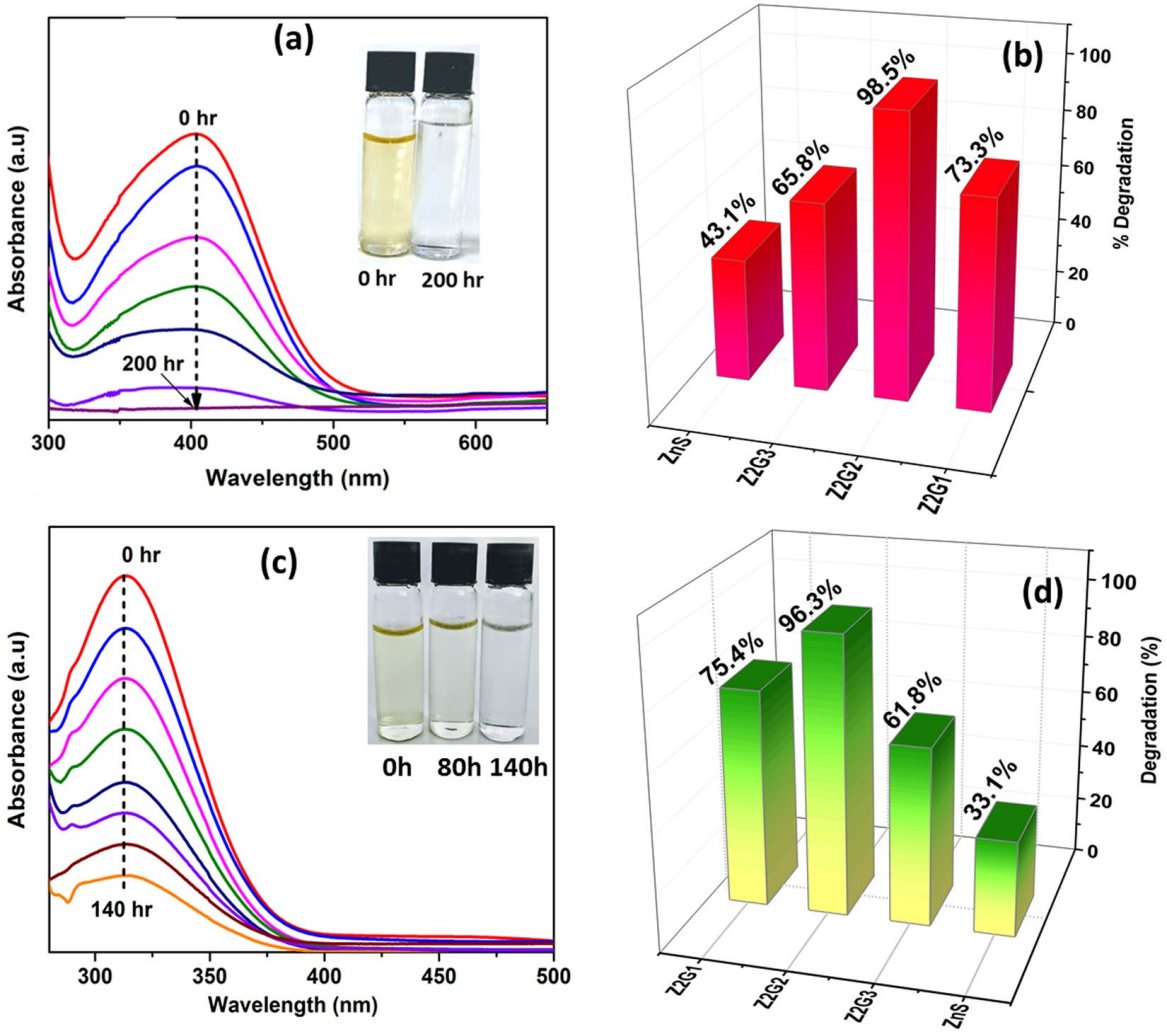
UV-Vis absorption spectra of Z2G-2 nanohybrid at different time intervals (**a**) 2-NP and (**c**) 4-NP MO (in-set discoloration of dyes. Photocatalytic degradation efficiency of Z2G nanohybrids towards 2-NP (**b**) and 4-NP (**d**).

In addition to the dyes, the photocatalytic degradation of highly toxic phenolic pollutants i.e 2-nitrophenol and 4-nitrophenol was also investigated using the fabricated heterostructured nano- photocatalysts as showed in Fig. 8. It can be seen that the decrease in characteristic spectral peak intensity of 2-nitrophenolate at 403 nm which gradually gone down (Fig. 8a) and discoloration of the phenolate (inset of Fig. 8a) with increasing irradiation time indicating the photodegradation of the nitrophenol. As shown in Fig. 8b, the hybrid Z2G-2 exhibits the best photocatalytic activity toward photocatalytic degradation of 2-nitrophenol, which is almost double the activity of nano ZnS and also higher compared to other Z2G nanohybrids after 200 min of visible light irradiation. Similarly, for 4-nitrophenol, the decrease of the peak intensity at 317 nm is clear evidence of its continuous effective degradation under visible light using this nanohybrid photocatalyst (Fig. 8c). The high degradation efficiency of 4-NP was noted for Z2G-2 nanohybrid. No additional reducing agents were used, which further confirm the photocatalytic degradation was attributed to a direct photo-hole oxidation and superoxide anions which are known to degrade and mineralize the phenolates under visible light irradiation^49^. Overall, the results confirm that at optimized composition of ZnS-ZnO heterostructure and graphene, owing to the synergistic effects fostered from individual components, the synthesized ZnS-ZnO/graphene nano-photocatalysts displayed significant effectiveness in removal of toxic pollutants from aqueous media.

**Figure 9:**
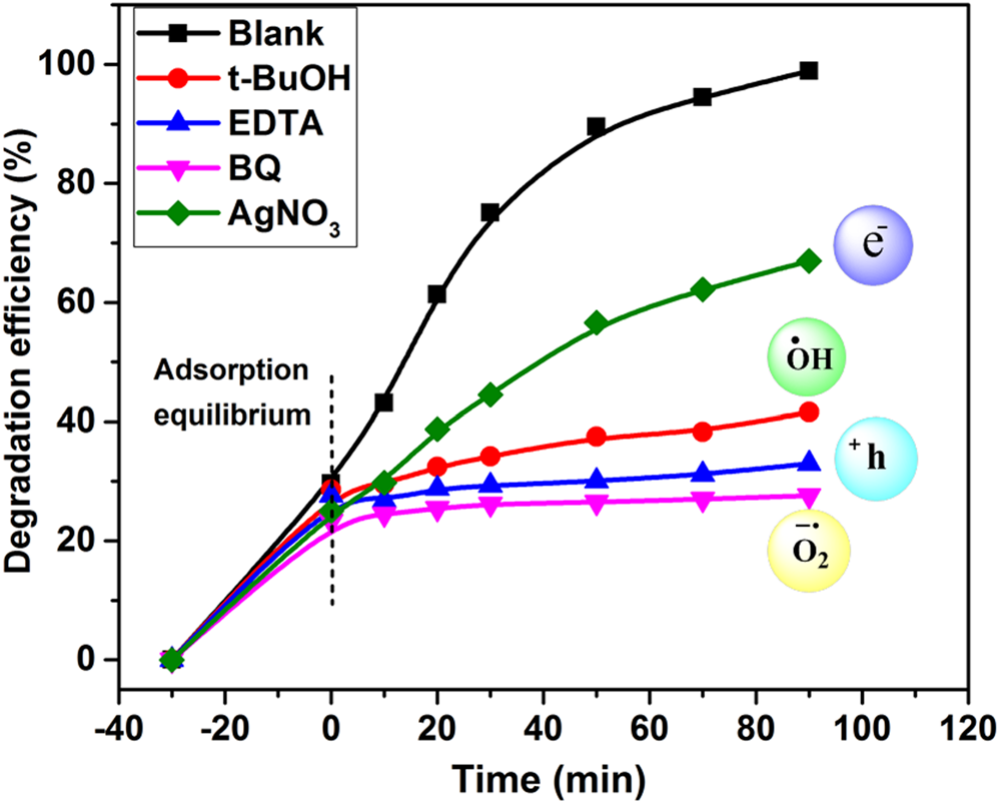
Scavenging experiment of active species during the photocatalytic degradation of MB over Z2G-2 nanohybrid under visible light irradiation.

For better understanding of the photocatalytic mechanism, it is important to investigate the active species formed during photocatalytic process. In general, photocatalytic degradation of dyes and phenols primarily comprises several active radical species which includes hole (h^+^), electrons (e^−^) superoxide anion radical (^●^O_2_^−^), and hydroxyl radicals (^●^OH)^50^. Hence, to distinguish the role of the reactive species, scavenging analysis were performed using series of scavengers during the photodegradation process of MB. Figure 9 shows the photocatalytic degradation of MB over Z2G-2 nanohybrid in the presence of different scavengers under visible-light irradiation. A remarkable decrease in photocatalytic activity of the Z2G-2 nanohybrids was observed in presence of BQ and EDTA-2Na scavengers, highlighting that the both photo-generated superoxide anion radicals and holes are the main oxidative species and play key roles in the photocatalytic process of MB under visible irradiation. In contrast, the photodegradation efficiency of MB is slightly affected in presence of AgNO_3_, indicating e^−^ are not the dominant active species in this process. Similarly, using t-BuOH, a moderate decrease in efficiency was recorded implying that the ^●^OH species plays adequate role in the degradation process.

Briefly, these scavenging experiment results point out that the photogenerated holes and ^●^OH species together with superoxide anion radicals makes the main oxidative force for photocatalytic degradation of the pollutants.

**Figure 10:**
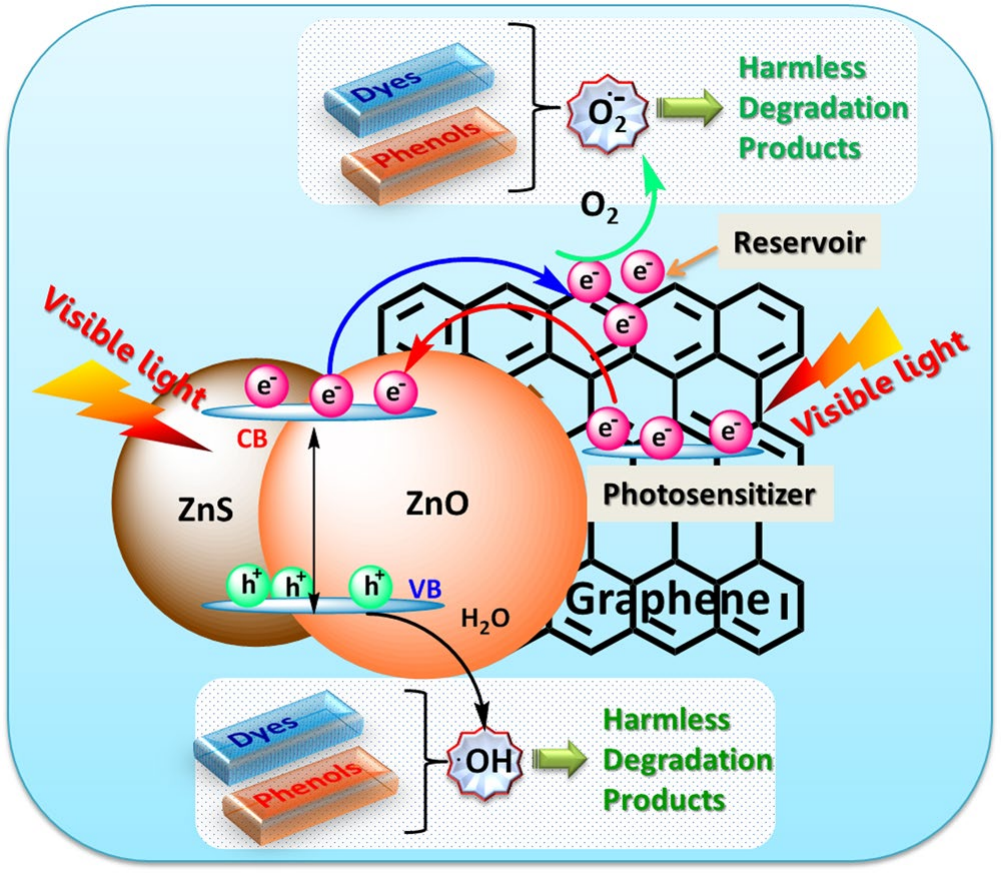
Schematic illustration of the charge separation and the transfer of photo-induced charge carriers in Z2G nanohybrids for MB and MO dye degradation under visible light irradiation.

Hence, considering the importance of the formation of reactive radical species during the photoexcitation process, the mechanism of photocatalytic dye degradation can be presented as shown in Fig. 10. Under photo-irradiation, the ZnS-ZnO heterostructures excite the electrons from its valence band (VB) state to the conduction band (CB) state and eventually form the electron–hole pairs. During this transformation, the VB is left with the positively charged holes which lead to the formation of hydroxyl radicals. Similarly, on other hand the electrons generated at conduction band (CB) are reactive enough to reduce the molecular oxygen. Hence, the resulting reactive radical species possesses powerful oxidizing properties which are highly effective in carrying out degradation of the dye molecules^51^. Also, the presence of graphene play dual yet pivotal role in enhancing the visible light photocatalytic activity of the ZnS-ZnO heterostructure. Firstly, by lowering the rate of electron–hole pair recombination through effective electron transfer from the CB of ZnS-ZnO to graphene reservoir through percolation mechanism^36^ and secondly by boosting the visible-light photocatalytic activity of ZnS-ZnO by photosensitization^30^. Under visible light irradiation, photo-excited electrons are generated from graphene and then transferred to the CB of ZnS-ZnO, which therefore transforms wide band gap ZnS-ZnO to a visible light photocatalyst. Such a photocatalytic reaction mechanism has also been proposed in previous semiconductor-graphene or carbon nanotube visible light photocatalysts^30,52^. Furthermore, bestowed with exceptional electron conductivity, the layered graphene network could promote the rapid mobility of the reactive charge carriers and consequent separation of the charge may occur. The photocatalytic degradation pathway and formation of intermediates for MB^53^, MO^54^, nitro-phenols^49^ have been extensively studied which indicates the final decomposition of these pollutants resulted into smaller harmless species such as water, CO_2_, NO_3_^−^, ONOO^−^ etc., respectively. Thus, owing to the above mentioned facts the as prepared Z2G nanohybrids display higher photocatalysis performance in decomposition of both anionic and cationic organic dyes and nitrophenols.

## Conclusions

A highly efficient, solvent-free and scalable method to prepare heterostructured nanohybrids of ZnS-ZnO/graphene using nontoxic and surfeit elemental sulfur as sulfidizing agent was reported. A series of nanohybrids with controllable ZnS:ZnO heterostructure composition and weight loading of graphene was *in-situ* fabricated and thoroughly characterized. The results verified that the nanohybrid composition includes nanosized cubic ZnS and wurtzite ZnO nanoparticles that are uniformly distributed within graphene matrix. Further, we firstly reported that, the content graphene precursor (GO) play key role in the controlling the ZnS:ZnO composition in the final nanohybrids. A remarkable photocatalytic activity was achieved for resultant nanohybrids under visible light and effective degradation of dyes (MB and MO) and nitro-phenol was accomplished, which underlined the versatility of the fabricated nano-photocatalysts. The enhanced photoactivity was ascribed to the lowered recombination rate for the photo-generated charge carriers, facile charge transfer and photo-sensitization-cum-strong dye adsorption due to the presence of graphene. Amongst nanohybrids, composition containing 10 wt% graphene and higher ZnO:ZnS ratio, showed superior photocatalytic activity which is due to the lower band gap, higher generation of electron–hole and their rapid transport. Overall, the present study offers greener pathway to fabricate ternary nanocomposites having tailored composition for wide range of applications.

## Methods

### Chemicals and materials

The following chemicals, graphite powder (Sigma–Aldrich, 10 mesh), sulfuric acid (Sigma–Aldrich, ACS reagent, 95.0–98.0%), hydrochloric acid (Sigma–Aldrich, ACS reagent, 37%), potassium permanganate (Fischer Scientific, ≥99%), hydrogen peroxide (Sigma–Aldrich, 30 wt% in H_2_O), sodium hydroxide (Sigma–Aldrich, ACS reagent, ≥97.0%), zinc acetate dehydrate (Sigma–Aldrich, ACS reagent, ≥98%) and phosphoric acid (Sigma–Aldrich, ACS reagent, ≥85 wt% in H_2_O). Scavengers p-benzoquinone (BQ), t-Butanol (TBA), ethylenediaminetetraacetic acid disodium salt (EDTA-2Na), and AgNO_3_ (all Sigma–Aldrich) were used as received. Zinc hydroxyacetate was prepared by procedure presented in our previous article^28^.

### Graphite oxide (GO) synthesis

A graphene precursor i.e graphite oxide was synthesized from oxidative exfoliation of the natural graphite by using improved Tour’s method^55^. Typically, sulfuric acid (200 ml) and phosphoric acid (25 ml) was simultaneously added to the 5 litter Erlenmeyer flask and mixture was cooled in ice-bath. To the cold acid mixture, the natural graphite (5 gm) was added and mixture was continuously stirred to achieve uniform dispersion of the flakes. Afterwards, potassium permanganate (2.7 gm) was added in portions over 30 min. To ensure the complete graphite oxidation, the final mixture was then continuously kept under stirring for 3 days at ambient temperature. The oxidation reaction was apparently evidenced by noticing the colour change from purplish green to dark brownish. Subsequently, the oxidation reaction was terminated by adding hydrogen peroxide (35%) solution, and immediately the colour of the reaction mixture was changed to golden yellow, demonstrating a high degree of graphite oxidation. The as –prepared GO dispersion was repeatedly washed using diluted hydrochloric acid (1 M) to remove the residual salts, metal ions and other impurities. Finally, the GO dispersion was subjected to the dialysis process in de-ionized water to attain stable pH value 6 to 7.

### Preparation of ZnS-ZnO/graphene nanohybrids (Z2G)

In typical procedure, 500 mg GO powder, stoichiometric quantity of zinc hydroxyacetate and elemental sulfur were ball milled using IKA ULTRA-TURRAX® Tube Drive control homogenizer at 4000 rpm for 30 min using 5 balls, each weighing 509.3 mg. Further, the resulting mixture was thermally treated in a tubular furnace at 400 °C for 2 hours with a constant heating rate of 5 °C min^−1^ under continuous argon flow to finally obtain ZnS-ZnO/graphene heterostructured nanohybrids (Scheme 1). Also, based on composition, the colour of the final hybrids was changed from light brown to greyish-black. A stoichiometric amount of GO was used in order to obtain composites with 5, 10, and 30 wt% graphene loading and abbreviated as Z2G-1, Z2G-2 and Z2G-3 respectively. For comparison, similar methodology was employed to prepare pristine ZnS nanoparticles.

### Characterization

As prepared samples of Z2G nanohybrid and ZnS nanoparticles were extensively characterized using different techniques. The X-ray powder diffraction (PXRD) analyses were conducted on a Philips X’Pert Pro X-Ray diffractometer equipped with a scintillation counter and Cu-Kα radiation reflection mode. The microscopic morphology and structures of the samples were characterized using a FEI Tecnai (G20) transmission electron microscope (TEM/HRTEM) and Zeiss (1540 XB) scanning electron microscope (SEM) coupled with energy dispersive X-ray analysis (EDX). The X-ray photoelectron spectra (XPS) were conducted by using a Surface Science Laboratories, Inc. (SSX-100) system equipped with a monochromated Al K_α_ X-ray source, a hemispherical sector analyzer (HSA) and a resistive anode detector. The specific surface area and porosity of the resulting Z2G nanohybrids were obtained using ASPS 2010 (Micromeritics, USA) Brunauer-Emmett-Teller (BET) nitrogen adsorption-desorption at liquid N_2_ temperature. The samples were pre-treated at 100 °C in a high vacuum for 24 h before N_2_ adsorption using a Quantachrome Autosorb gas-sorption system. The Raman spectra of the Z2G nanohybrids were measured using a Raman microscope (LabRam HR, Horiba Scientific) with an excitation wavelength of 633 nm. The optical absorbance spectroscopy in absorbance and diffuse reflectance mode was recorded using Shimadzu UV-vis-NIR spectrophotometer (Model UV-3600) over a wavelength range of 200 to 800 nm. Room temperature photoluminescence spectra were obtained from Shimadzu spectrophotometer (RF-5301PC), and the excitation wavelength was 350 nm. The elemental analysis was performed on EuroVector (EUROEA3000) CHNS element analyser. UV-visible absorption spectrum was recorded on Cary 500 UV-vis spectrophotometer (Varian, Palo Alto, CA). The ball milling performed on IKA ULTRA-TURRAX® Tube Drive control homogenizer using five stainless steel balls, each weighing 509.3 mg.

### Photocatalytic activity

First, the as-prepared photocatalyst samples (10 mg) were dispersed in 100 mL deionized water using ultra-sonication. Later, 100 mL of aqueous solution of the respective dyes and nitro phenol (1 × 10^−5^ mol L^−1^) was added to the catalyst dispersion under continuous stirring. The resultant dispersed solution was then stirred in the dark in order to achieve the adsorption–desorption equilibrium for the photocatalysts, dye and dissolved oxygen. Consequently, the suspension was exposed under a visible light source (400 W metal halide lamp with spectral range 420–700 nm, OSRAM, Germany). The reaction flask was mounted in such way that the incident light was directed to the surface of the solution at a distance of 10 cm. 3 mL aliquots of the suspension were taken at regular irradiation intervals and centrifuged to separate the photocatalyst. The absorption of the clear supernatant was monitored using a UV-Vis spectrometer. The concentration of the remnant contaminants was determined by recording the changes in the absorbance of solutions at maximum of 664 nm, 460 nm and 403 nm for MB, MO and phenolates, respectively during the photocatalytic process.

The efficiency of photodegradation in percentage is calculated from the equation (4) given below:

4 $$ \% \,Photodegradation\,efficiency={\rm{X}}\frac{{C}_{0}-Ct}{{C}_{0}}100$$

where C_0_ = initial dye concentration and C_t_ = concentration of dye after photodegradation at time ‘t’.

For the radical scavengers experiments, 10 mg of photocatalyst was dispersed into 50 mL of the MB (10 ppm) solution, and then 20 mg of p-benzoquinone (BQ), t-Butanol (TBA), ethylenediaminetetraacetic acid disodium salt (EDTA-2Na), and AgNO_3_ were used as superoxide anion radical (^●^O_2_^−^), ^●^OH scavenger, h^+^ (holes) scavenger, and e^−^ scavenger, respectively. Before irradiation, the solution was continuously stirred for 30 min in the dark to achieve absorption-desorption equilibrium and subsequently irradiated. An aliquot of 5 mL solution was withdrawn and analyzed with a UV-Vis spectrophotometer to measure its absorbance.

## Electronic supplementary material

Supplementary Information
