# Supplementary material for: Facile and scalable production of heterostructured ZnS-ZnO/Graphene nano-photocatalysts for environmental remediation
Source: Sci Rep. 2018 Sep 7;8:13401. doi: 10.1038/s41598-018-31539-7 (PMC6128855; doi:10.1038/s41598-018-31539-7)
Supplement: Supplementary file 1 — Supplementary Information [file 41598_2018_31539_MOESM1_ESM.docx]

**Supporting Information**

Facile and scalable production of heterostructured ZnS-ZnO/Graphene nano-photocatalysts for environmental remediation

Sunil P. Lonkar*, Vishnu V. Pillai and Saeed M. Alhassan*

Department of Chemical Engineering, Khalifa University, P.O box 2533, Abu Dhabi, UAE

E-mail: <saeed.alkhazraji@ku.ac.ae>; sunil.lonkar@ku.ac.ae

Tel:+971-26075944, Fax: +971-26075200

| **** |
| --- |
| Fig.S1 XRD pattern of GO and thermally reduced GO (graphene) |

| **** |
| --- |
| Fig.S2 Schematic representation showing the effect of GO content on formation of ZnS:ZnO heterostructure and their composition |

| **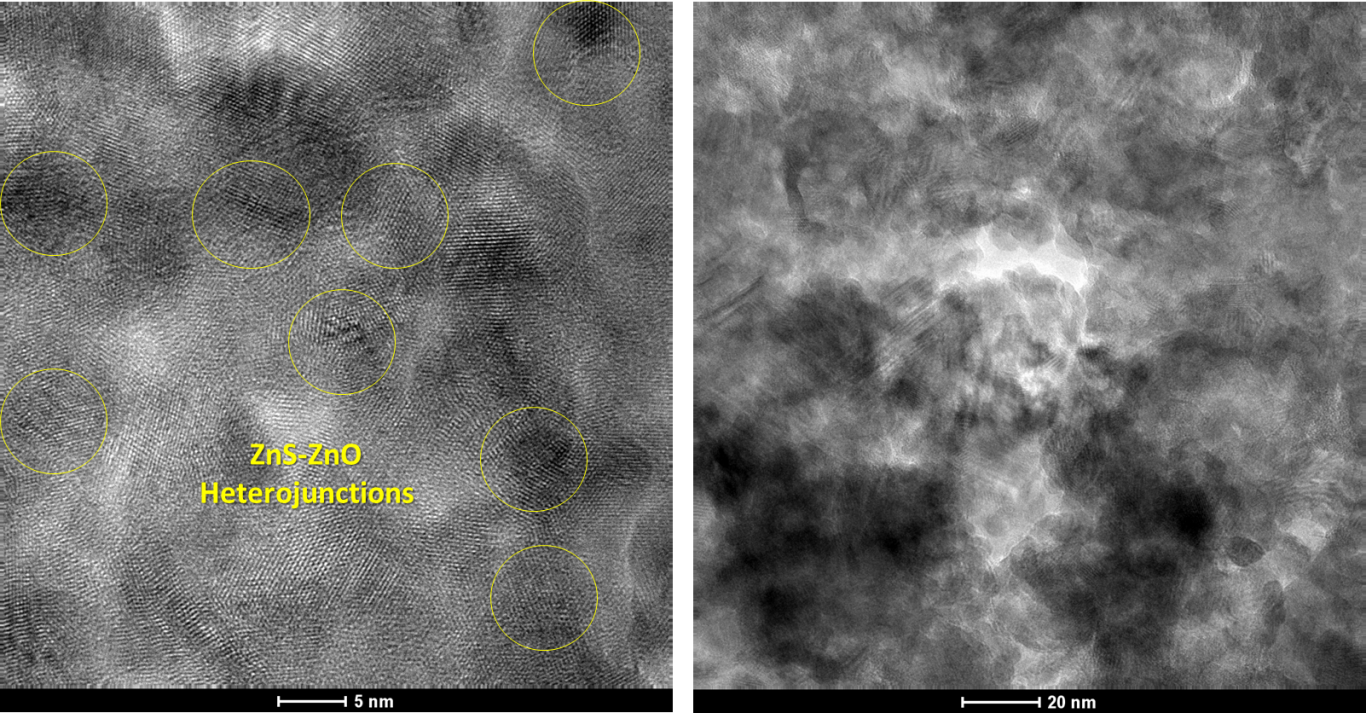** |
| --- |
| Fig.S3 Low-magnification TEM image of Z2G-1 nanohybrid. |

| **** |
| --- |
| **** |
| Fig.S4. SEM image (a), EDS spectrum (b) and corresponding elemental mapping images for Zinc, Sulfur, Oxygen and Carbon (c), respectively (c) for Z2G-2 (A) and Z2G-3 (B) nanohybrids, respectively.. |

| **** |
| --- |
| Fig.S5. XPS spectroscopy of GO (a) survey spectrum and (b) high resolution scan of carbon C1s. |

| **** |
| --- |
| Figure S6. Raman-scattering spectra of (a) Z2G-1 and (b) Z2G-3 nanohybrids |

|  |
| --- |
| Fig.S7. The graph of ln(C_0_/C_t_) verses irradiation time for Z2G nanohybrids and ZnS nanoparticles during MB dye photodegradation. |

|  |
| --- |
| Fig.S8. The graph of ln(C_0_/C_t_) verses irradiation time for Z2G nanohybrids and ZnS nanoparticles during MO dye photodegradation. |

|  |
| --- |
| Fig.S9. The recycling tests for resulting nano-photocatalyst during the photodegradation of MB for 90 min and MO for 160 min. |
